# Optimal vaccination schedule search using genetic algorithm over MPI technology

**DOI:** 10.1186/1472-6947-12-129

**Published:** 2012-11-13

**Authors:** Cristiano Calonaci, Ferdinando Chiacchio, Francesco Pappalardo

**Affiliations:** 1, CINECA, Bologna, Italy; 2, University of Catania, Catania, Italy

**Keywords:** Cancer, Optimization, Artificial intelligence, High performance computing

## Abstract

**Background:**

Immunological strategies that achieve the prevention of tumor growth are based on the presumption that the immune system, if triggered before tumor onset, could be able to defend from specific cancers. In supporting this assertion, in the last decade active immunization approaches prevented some virus-related cancers in humans. An immunopreventive cell vaccine for the non-virus-related human breast cancer has been recently developed. This vaccine, called Triplex, targets the HER-2-neu oncogene in HER-2/neu transgenic mice and has shown to almost completely prevent HER-2/neu-driven mammary carcinogenesis when administered with an intensive and life-long schedule.

**Methods:**

To better understand the preventive efficacy of the Triplex vaccine in reduced schedules we employed a computational approach. The computer model developed allowed us to test in silico specific vaccination schedules in the quest for optimality. Specifically here we present a parallel genetic algorithm able to suggest optimal vaccination schedule.

**Results & Conclusions:**

The enormous complexity of combinatorial space to be explored makes this approach the only possible one. The suggested schedule was then tested in vivo, giving good results. Finally, biologically relevant outcomes of optimization are presented.

## Background

The role of the immune system in tumor surveillance is today clearly established, and tumor immunologists are actively working to devise preventive and therapeutical vaccines against cancer. Living organisms are natural complex systems and modeling may play a crucial role since models can also be built with approximate and imperfect knowledge of the phenomenon, and model parameters (initial data, entities, relations between entities) can be adjusted to fit modeling results to experimental measurements [1].

Cancer immunoprevention is a recent development of tumor immunology that aims at preventing tumor onset with immunological means, in particular vaccines. The main challenge issuing from successful experiments in genetically-modified mice is now to translate immunoprevention to human situations. In this way, once the vaccine has been demonstrated to be effective in preventing the targeted tumor, it is necessary to find an optimal vaccination schedule that minimizes both the administrations of the vaccine and the eventually present side effects. Obviously the time and the costs needed for an exhaustive search are prohibitive [2].

The evaluation of the antitumor efficacy of cancer vaccines in mouse models (also here referred to as *biological models*) is a required prelude to the clinical use of these treatments. Testing of some cancer vaccine features, such as the best conditions for vaccine administration, can be very difficult or even impossible only through experiments with biological models simply because a high number of variables need to be considered at the same time. This is where computational models can prove handy as they have shown to be able to reproduce enough biological complexity to be of use in suggesting new experiments [3,4]. This characteristic makes computer models suited to perform “what-if” analyses to elucidate relationships between different phenomena and to aid in the validation or rejection of working hypotheses. Indeed, computational models can be used in *addition* to biological models.

We developed an agent based model (ABM) of the effects of a vaccine designed to prevent mammary carcinoma in transgenic mice [5]. This model faithfully summarizes not only the outcome of vaccination experiments, but also the dynamics of immune responses elicited by the vaccine [6-10].

We then used a parallel genetic algorithm to search for an optimal vaccination schedule. The predicted schedules were tested *in vivo*, giving good results [11]. The approach plays the role of a virtual laboratory performing in a few days *in silico* experiments that would take years *in vivo*.

In order to speed up the search for an optimal vaccination schedule, our genetic algorithm is parallelized using Message Passing Interface (MPI). Furthermore, an improved master-slaves approach enabled us to examine high performance measurements in terms of program execution time and load balancing.

The plan of the paper is the following. Firstly we briefly introduce the complexity of the biomedical system (interactions of immunity, vaccine and cancer); then we explain the motivation that leaded to the use of parallel computing. Section “The informatics infrastructure” briefly describes the core of the vaccine protocols evaluators and gives a formal definition of the optimization problem. Section “Parallelization” briefly introduces the definition of parallelization in computer science. Section “Parallel genetic algorithm” gives the details on the implementation of the parallel genetic algorithm; section “Results of PGA over MPI” presents the benchmarks of the approach and experimentally proves the good performance of the algorithm. Finally in Section “Discussion and Conclusions” we give our final considerations, highlighting the biologically relevant outcomes of optimization.

For the sake of completeness, we briefly introduce in this section the main features of the human immune system and the basic concepts of tumor immunology and cancer vaccines. Moreover, we focus on the potential of a special vaccine tested on HER-2/neu transgenic mice.

### The immune system

The immune system responds to molecules identified as foreign (mainly components of microbes) to prevent infectious diseases, by various mechanisms altogether named immune response [12]. A first line of defense of the immune system is supported by the innate immunity that includes physical barriers, soluble mediators and specialized killer cells. The innate immune response remains essentially unaltered by repeated infections. The adaptive immune system provides a second line of defense against infections as it recognizes in a specific way distinct components called antigens. Lymphocytes are the cellular players of this elaborated response and are able to store information on the acquired antigen recognition, to improve the immune response to repeated exposures. Finally, the adaptive immunity is specific for foreign antigens and tolerant to autologous (self) components.

The lymphocytes population includes millions of clones, each one with a different specific antigen receptor. This variability among lymphocytes receptors is the reason for lymphocytes ability to recognize a high number of different antigens. Lymphocytes are mainly divided in T and B cells, and bear antigen receptor molecules on their cell surface. All of these specialized cells and parts of the immune system offer the body protection against disease. This protection is called immunity.

### Tumor immunology

Several clinical and preclinical studies highlighted a strong correlation between immune system weakness and disorderly cell growth. The immune system physiologically prevents tumor onset, but the incidence of neoplastic diseases proves that cancer immune surveillance is not completely effective. Reasons for tumor progression could be related to transient immunodepression, reduced efficacy of the immune system response with aging and tumor cell acquisition of the capability to exploit immunological mechanisms and evade immune surveillance [13-15].

Immune attack made in response to tumors is moved by both innate and adaptive immunity, including many molecules and cellular entities that act together and in a cooperative way in order to limit cancer growth. Briefly, phagocytes (granulocytes and macrophages), actors of the innate immunity, directly destroy tumor cells and produce cell fragments. Antigen presenting cells (APC) pick up and process these fragments ultimately presenting tumor antigens for lymphocyte recognition. Dendritic cells, which are professional APCs, uptake tumor antigens in the periphery then migrate to lymph nodes. Moreover, natural killer (NK) cells kill tumor cells with a low MHC expression and play a key role in the defense against circulating metastatic cells.

The T helper cell population is the play-maker of the adaptive immunity team against tumors. Th cells, activated by antigen recognition on APCs, proliferate and activate, by cytokine secretion, Tc, phagocytes, NK cells and B cells. Most solid tumors are protected from antibody or complement dependent lysis, consequently in the antitumor immune response the role of B cells is (mistakenly) considered marginal. Moreover B cells can even downregulate T cell responses promoting tumor growth. Finally also Treg cells can inhibit antitumor responses [16].

### Cancer vaccines

The idea of developing strategies to support the immune system against tumors has been producing several immunological approaches effectively able to limit tumor growth. These strategies can be passive as monoclonal antibodies administration, or active as vaccines [17]. The cure of established tumor masses by immunological strategies (immunotherapy) has produced poor results suggesting to address efforts to adequately stimulate immune system before tumor onset (immunoprevention), to protect the organism from specific cancers. Preclinical studies have shown that prevention is more effective than cure in the tumor immunology field [16].

Cancer vaccines actively enhance a specific immune response against target tumor antigens. Tumor antigens include a huge number of tumor-associated molecules mostly recognized by the immune system of the host as self, as they are also expressed by normal cells [18]. Consequently, a successful antitumor immune response against such self antigens requires to break the immune tolerance. Among many described tumor antigens, only a few molecules proved to be good target antigens. Tumor associated molecules that are essential for tumor growth and progression could be suitable cancer vaccine targets, since they cannot be easily downmodulated or negatively selected in precancerous lesions under the pressure of a specific immune attack. Lollini and colleagues have defined these molecules as *oncoantigens*[16,19].

### Cancer immunoprevention in HER-2/neu transgenic mice

The human epidermal growth factor receptor 2 (referred to as HER-2 or ErbB2) is a membrane tyrosine kinase overexpressed in 25-30% of human breast cancers [20]. HER-2 has been widely used as target for immunopreventive strategies often evaluated against mammary carcinogenesis in rat HER-2/neu transgenic mice. A large number of studies have found treatments able to delay and/or reduce tumor onset up to a complete protection [19,21].

The Triplex cellular vaccine is one of the most effective preclinical preventive vaccine [19]. The vaccine is called Triplex because it has three main components: the target antigen, HER-2/neu, and two adjuvant stimuli, IL-12 and allogeneic MHC molecules. IL-12 is needed to improve antigen presentation and consequently increase Th cell activation. Allogeneic MHC molecules are relevant to break the tolerance to HER-2/neu self antigen by stimulating multiple T cell clones and causing a broad production of immunostimulatory cytokines [17,22]. Mice were completely protected from mammary tumor onset by repeated administrations of the Triplex vaccine, starting at an early age (6 weeks of age). Untreated mice had multiple mammary carcinomas at six months of age while almost all vaccinated mice were tumor-free at one year of age doubling the life expectancy of these mice.

## Methods & Results

The efficacy of the Triplex vaccine was related to the number and distribution of administrations along the mouse life. The Triplex vaccine was administered in mice according to *chronic* protocol based on 4-week vaccination cycles, starting from 6 week of age for the entire lifetime of mouse or until one year of age, at least. Mice received four vaccine administrations over the first 2 weeks of each 4-week cycle [22]. Such a high number of vaccinations actually limits the clinical use of the vaccine because it reduces patients compliance and increases the risk of side effects. Only three vaccination cycles were insufficient [23].

The efficacy of a treatment is strongly dependent on its dosage and schedule of administration. Many factors have to be considered in defining a new treatment schedule. Individual diversity and risk of side effects must be taken into account. If the former has effects on the minimal (lower bound) dosage of a treatment, the latter establishes an upper bound on the maximum allowed dosage to avoid side effects such as toxicity. To determine the schedule of a new treatment the common practice is to make use of the *medical consensus*, a typical policy where a representative group of experts in some medical areas commonly define the guidelines for the administration of a treatment, basing their decision on the state-of-the-art knowledge and past experimental evidence. It is worth to note that in vivo research focused in finding better vaccination protocols was discouraging, since it would require many sets of experiments in vivo, each lasting one year, with prohibitive costs.

It is now clear that the availability of a computational methodology that helps biomedical scientists to define optimized vaccination schemes would be very useful.

### The informatics infrastructure

To tackle the problem of determining if better vaccination protocols for the Triplex vaccine exist, we developed an in silico computational model (named SimTriplex) specifically designed to reproduce the effects of Triplex vaccine against the development of mammary carcinoma in HER-2/neu transgenic mice [5,24-26]. SimTriplex models all various classes of immune functional activity, phagocytosis, immune activation, opsonization, infection, cytotoxicity and specific/aspecific recognition. They are described using probability functions and translated into computational rules. An interaction between two entities is a complex stochastic event which may end with a state change of one or both entities. Interactions can be *specific* or *aspecific*. Specific interactions need a *recognition phase* between the two entities (e.g. B ⇔ TAA); recognition is based on Hamming distance and affinity function and is eventually enhanced by adjuvants. We refers to *positive interaction* when this first phase occurs successfully. Aspecific interaction do not have a recognition phase (e.g. DC ⇔ TAA). When two entities, which may interact, lie in the same lattice site then they interact with a probabilistic law. Both specific and aspecific interactions are stochastically determined using a probability function, which depends from different parameters, computed via random number generators. Changing the seed of the random number generator one gets a different sequence of probabilistic events. This simulate the biological differences between individuals who share the same events probabilities. In order to model the continuous carcinogenic process of HER-2/neu transgenic mice, newborn tumor cells appear at each time step and are randomly placed on the lattice, whereas existing tumor cells duplicate. The simulation runs for a number of steps, typically equivalent to more than 1 year of real time. If the total number of tumor cells exceeds a given threshold, which indicates the formation of a palpable tumor mass, the simulation is stopped. Individual diversity observed in the experimental set-up is simulated through the use of pseudo-random number generators. Pseudo-random numbers affect the outcomes of various probabilistic events at starting of the simulation (e.g. entities initial position in the lattice) as well as all the events that happen during the simulation, such as the order and outcomes of interactions. Each run of the simulator initialized with a given random number thus represents a *virtual mouse*. Experimental variability among mice is given by the use of different seeds for the pseudo-random number generator.

#### The optimal vaccination schedule search problem

An optimal schedule maintains its efficacy with a minimum number of vaccine administrations. As in standard drug administration, the vaccine has to be effective for a high percentage of patients. In lack of quantitative methods, this is usually achieved using *medical consensus*, i.e. a public statement on a particular aspect of medical knowledge available at the time it was written, and that is generally agreed upon as the evidence-based, state-of-the-art (or state-of-science) knowledge by a representative group of experts in that area. Our goal is therefore to have a quantitative approach, using simulators and optimization techniques, that can help biologists in designing vaccine protocols. It is worth to mention here the fundamental definitions of the optimization problem we will deal with. Let us consider a time interval [0,*T*], in which we study the action of the vaccine on a set of virtual mice *S*. This can be, for example, the time-length of the *in vivo* experiment. We then discretize the given time interval in *N*−1 equally spaced subintervals of width *Δt*, i.e. {*t*_1_=0, *t*_2_, …,*t*_*i*_, …, *t*_*N*_=*T*}. The time interval *Δt*corresponds to the time of possible vaccine administrations, e.g. every 8 hours.

Let **x**={*x*_1_,*x*_2_,…,*x*_*i*_,…*x*_*N*_} be a binary vector representing the sequence of vaccine schedule where *x*_*i*_ = 0/1 means respectively administration/no administration of the same quantity of vaccine at time *t*_*i*_. The number of vaccine administrations is given by $\left(n=\overset{N}{\underset{i=1}{\sum }}x_{i}\right)$. The search space *D* for this problem has therefore cardinality 2^*N*^. For *T* = 400 days, and *Δt* = 24 hours the cardinality is 2^400^ which prevents any chance of an exhaustive search. Anyway, one wet biologists requirement is that vaccine administrations can be performed only twice a week (monday and thursday) and this is already considered a very intensive vaccination schedule from an immunological point of view. Luckily, this greatly reduces the cardinality of the search space *D*, from 2^400^(∼10^120^) to 2^114^(∼10^34^).

To conclude, it is needed an optimization technique that can deal with this kind of complexity in reasonable time. In the following we will describe in details what we developed.

### Parallelization

In the informatics field, parallelization is the activity that permits to exploit several computing resources working at the same time (in parallel), in order to speed up a computational process. With the increasing of the computational power, parallelization is gaining more interest and several kind of solutions are now available, from the multicore processors mounted in commercial computers to more complex distributed architecture made up of several machines.

There are two important aspects to consider when thinking to use the parallel approach: 

● parallelization is not the solution for any type of application looking for a speed up,

● parallel architectures do not improve the performance of traditional algorithms written to run on a single processor.

For what concerns the first statement, it is quite simple verifying whether or not a complex process can benefit of parallelization: if the tasks that constitute the process can be performed independently parallelization is possible. But parallelization is not the merely use of a complex hardware structure with a high number of CPUs because, in order to exploit all the potentiality of such architectures, programmers have to write a parallel code that can be distributed and processed by independent resources. Moreover, it is worth to highlight that the term resource can refer both to a hardware and a software entity that participates to the run of a program. The former resources are generally the CPU and the different type of memory (like registers, RAM and hard disk) while the latter concern the status of the program in terms of data - which are generally stored in the dynamic memories for the runtime execution (registers and RAM). Therefore, when dealing with parallelization both the hardware and the software play an important role for the execution of a code.

In general there are two main approaches for parallelization and the best choice depends on the type of problem to solve: 

1. break up a complex routine into its subroutines and distribute them to different CPUs; in this case CPUs can access to a shared space of read-only memory in order to perform computations and have to synchronize if a subroutine needs the results of another subroutine;

2. if the process consist of completely independent routines, parallelization can be performed distributing these independent routines to different CPUs which synchronize their resources only at the begin and the end of the process.

While those applications that make use of the first approach could, at limit, turn back into a sequential run (when any subroutine need the results of the previous one), iterative simulations represent a good example of algorithm that can be solved using the second approach; in this case, ideally, the speed up increases linearly with the increasing of the number of CPUs.

For what concerns our optimization problem, among all tried different strategies used, the successful one was represented by the use of genetic algorithm (GA) like described in [16]. GA [27-29] is a search heuristic methods used for the resolution of NP-complete problems characterized by the evaluation of a large number of possible solutions (population) through a fitness function. For this reason, GA can be seen as an iterative algorithm which - at any generation (iteration) - selects the best set among all the available solutions of the population, in order to perform the evolutionary process and find out new and more suitable solution of the problem.

#### Parallel genetic algorithm

The GA used in [11] discovered a vaccination schedules able to avoid solid tumor formation. These results have encouraged the application and the improvement of the GA for the seek of the optimum vaccination protocol as, at the state of the art, the mentioned vaccination schedules did not prevent the cancer cells to reach an unsafe growth level and, moreover, did not protect a large percentage of our population of *virtual* mice. Having this in mind, we modified the GA in order to constrain the research of the optimum vaccination schedule on a more strict biological basis that consider the protection of a *large class* of individuals and not only a single mouse. For this reason, we extended the population of mice and applied the same vaccination schedules to 8 randomly chosen mice at the same time.

The GA has been implemented as in [30] with a simulator that acts as fitness function evaluator for the individuals of the population, representing the possible vaccine schedules (or therapies). A GA with an attached simulator is a long and complex computational task and it requires a prohibitive amount of running time on a single CPU machine. Since, our GA strategy is based on a population of 80 individuals to be applied to 8 virtual mice for 150 generations, a run on a single CPU machine would require 8·80·150·*T*_*fit*_, where *T*_*fit*_ is the time needed to evaluate the fitness function, i.e. to run the simulator. In the previous equation, the only variable term is *T*_*fit*_, as it depends on the performance of the hardware. If we suppose that *T*_*fit*_ in a traditional single CPU machine is about 30 seconds, the previous simulation scenario will take about 32 days.

**Figure 1:**
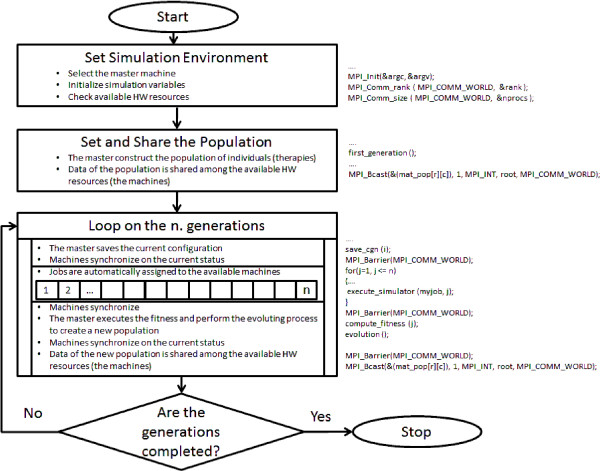
PGA flowchart.

According to the parallel computing approaches described in the previous section, the parallel version of the GA (PGA) can be implemented as shown in Figure 1. Due to the nature of this algorithm, the MPI technology was used as it offers a complete set of library for the automatic distribution of the work loads to the pool of the machines available and the synchronization of the parallel jobs. When adopting the MPI protocol all the machines involved receive the same piece of code but, thanks to the function of the MPI library, they can run selected parts of it. In particular, MPI functions permit to automatically define a machine as the master which is in charge to execute the sequential parts of the code and configure all the simulation environment(see the operations sketched in the *Set Simulation Environment*); moreover the MPI functions will detect all the other resources allowing the master machine to synchronize them with the simulation data to use for the parallel jobs.

For what concerns our PGA, as it is shown in Figure 1, the algorithm consists of the following sequential steps 

*Set Simulation Environment* and *Set and Share the Population*, for the configuration and the synchronization of the machines and

a pure parallel segment of code inside the *Loop on the n. generations* where the discovered available resources are automatically configured to run assigned pieces of simulation (*execute simulator*) in parallel.

At first, as a traditional GA, the master machine will configure the first generation of individuals that are passed to the other machines through the MPI function *MPI*_*Bcast*(·). After that, the resources synchronize *MPI*_*Barrier*(·) and start their scheduled jobs in parallel. Once all the machines have finished and their computations synchronized, the Master collects and organizes these results in order to compute the fitness function and start the process of evolution for the enhancement of the population. The process will continue until the number of generations over.

Once parallelism is incorporated into the GA, significant amounts of time can be saved. These results are significant also from a biological point, both in terms of time, mice and costs perspectives; they can be appreciated looking Table 1: the virtual laboratory implemented permitted to enlarge the number of therapies from 16 to 128, showing that it was possible to test a large amount of vaccine schedules within a reasonable period of time.

**Table 1:** Running time over different number of processors and therapies

**#CPU**	**# of therapies**	**time per generation (mins)**
4	16	7.667
8	16	4.955
16	16	3.503
32	16	3.560
64	16	3.525
4	32	16.824
8	32	9.715
16	32	6.488
32	32	5.261
64	32	4.639
4	64	39.663
8	64	28.804
16	64	15.069
32	64	7.072
64	64	4.345
128	64	4.486
16	128	21.591
32	128	11.975
64	128	7.862
128	128	5.158
256	128	4.997

The number of virtual mice are always set to 8

#### Results of PGA over MPI

**Figure 2:**
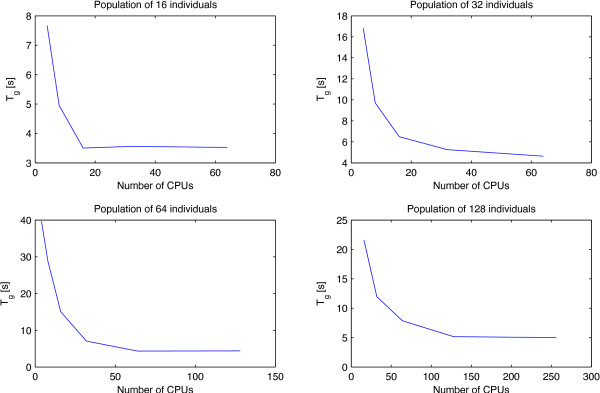
**Results of the simulations of Table **1. It shows the linear decreasing of the *T*_*g*_(time expressed in minutes) due to the usage of an increasing number of CPUs. These set of data were collected fixing the number of individuals of the population (from 16 to 128) scaling the number of CPUs from 4 to 256.

Figure 2 depicts the results of the simulations of Table 1, providing other interesting insights; in fact, it shows the linear decreasing of the *T*_*g*_(the time needed to perform one genetic algorithm generation), due to the usage of an increasing number of CPUs. These set of data were collected fixing the number of individuals of the population (from 16 to 128) scaling the number of CPUs from 4 to 256. For instance, let us to consider the top frame on the left of Figure 2, corresponding to the simulations of the PGA with 16 individuals; according to the results of Table 1, the time to perform one generation using 4 CPUs is about 7.66 minutes and doubling the number of CPUs to 8 the time needed decreases to 4.95 minutes. Again, if we double to 16 the number of CPUs the time of *T*_*g*_ keeps lowering to 3.5 minutes, confirming that it follows a negative slope typical of a linear function.

The same trend appears also looking at the other graphics and help to observe that the slopes of the curves become tinier to a threshold which indicates the lower bound for any *T*_*g*_(from 16 to 128 individuals), no matter the number of CPUs exploited. This result does not have to surprise and suggests some important considerations; in fact, on one side they confirm the effectiveness of the approach, showing the way how acting on the number of CPUs it is possible to scale linearly the time of computation but, above all, they reveal what is the bottleneck of the algorithm beyond which the increasing of CPUs do not improve the performance of the computation. This limit is related to the contribute of 

1. the waiting time due to the operations of synchronization among the CPU slaves and

2. the time of computation performed by the master CPU, at the beginning of a new generation, for the construction of the new individuals of the PGA population (the vaccine schedules).

**Figure 3:**
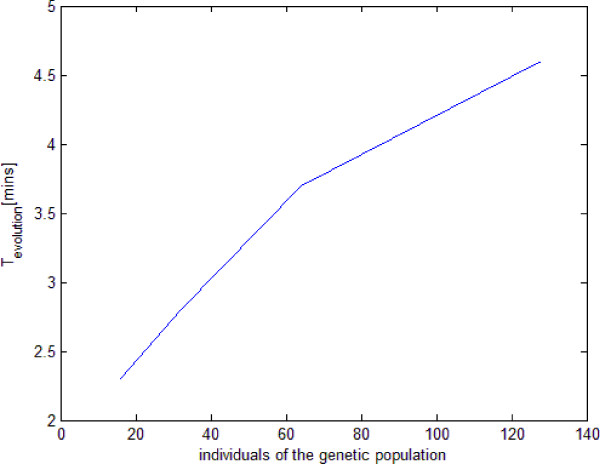
Time (in minutes) for the master CPU to process a new genetic population; it tends to increase with the number of individuals.

As it is highlighted in Table 1 this limit tends to increase from about 3.5 minutes for 16 individuals (using up to 16 CPUs) to 5 minutes for 128 individuals (using up to 128 CPUs). The reason why it cannot be lowered is that the operations of evolutions for the *i*^*th*^generation (i.e., the updating of new individuals at the beginning of a generation, typical of a genetic algorithm) cannot be parallelized and have to be performed always by the master CPU, once it has collected all the resulting data of the (*i*−1)^*th*^generation coming from the slave CPUs; this result is highlighted in Figure 3, showing how the time for the master CPU to process a new genetic population tends to increase with the number of individuals.

## Discussion and Conclusions

Optimization theory has a long tradition and the techniques are numerous. Most of the practical problems in physics, engineering and applied mathematics can be formulated as optimization problems. From this perspective the search for an optimized therapeutic protocol for the administration of a vaccine is no exception. In this article we have tried to show how this search for the best vaccine administration in terms of dosage and timings can be formulated as an optimization problem and then, how it can be solved using well-known well known artificial intelligence methodologies over supercomputing infrastructures.

The quality of the optimized protocol is strictly related to the goodness of the model. In particular the direct or indirect effects of the therapeutic agents on the malignancy need to be carefully taken into account since the optimization algorithms rely on a scoring method that price the solutions on the basis of their effects. Having this said, it appears clear that the more sophisticated and detailed the model is, the higher the chances to obtain an efficacious effect of the optimized therapy once it goes to the test bed.

We started from a real question: is it possible to reduce the number of Triplex vaccine administrations and maintain a high preventive efficacy? This is a typical clinical question that. The work we have described in this article showed that modeling is a concrete tool for study of cancer immunopreventive strategies and therefore can help in answering that question in the biomedical world.

From the biomedical point of view, the main outcomes of the suggested protocol, after its long term in vivo validation, can be summarized as following. As predicted in silico, many vaccinations of the Chronic protocol are redundant and can be avoided. A rapid priming of young mice is required for long-term protection from tumor onset, and the accuracy of mathematical modeling of early immune responses is critical. Finally, the model should take into account the ageing of the immune system. As presented in [11], the protocol of vaccination should be revised in the elderly hosts.

In this paper, we presented a parallel framework to execute a genetic algorithm that uses a simulator as a fitness evaluator. It suggested near optimal vaccination schedule that was then tried in vivo. The run of this kind of genetic algorithm would have required about 32 days for a single run. We then implemented the genetic algorithm over MPI technology. The main problem to be deal with was the linear scaling of the implementation. We used a master-slave approach that allowed us to have a good linearity scaling, optimizing the use of the available CPUs.

Parallel computing was successfully applied in drug optimization, leading to the development of a real virtual lab to analyze and optimize vaccine protocol administrations.

It is worth to mention that, in the view of real possible applications in biomedical informatics, for example in hospitals, it is unlikely that clinics or hospitals own HPC infrastructure dedicated to a virtual lab. However nowadays computer science technology advances created HPC systems (think about multi-cores computers) that can fit in a doctor’s room.

To conclude, we can not forget what is the other side of the coin. Models are simplifications of reality and as a such can leave apart important aspects of the phenomenon under study. A good model needs to be based on clinical or preclinical data and its improvement needs to make use of available clinical and preclinical testing of predicted data. Only through a carefully checked adherence with reality we can hope that our model produces useful meaningful biological knowledge. Perhaps not perfectly accurate but at least instructive.

## Competing interests

The authors declare that they have no competing interests.

## Authors’ contributions

CC & FP developed the MPI architecture and wrote the code. FC makes the in silico experiments and performed the benchmarks. FP supervised the project. All authors read and approved the final manuscript.

## Pre-publication history

The pre-publication history for this paper can be accessed here:

http://www.biomedcentral.com/1472-6947/12/129/prepub
